# *Plandemic* Revisited: A Product of Planned Disinformation Amplifying the COVID-19 “infodemic”

**DOI:** 10.3389/fpubh.2021.649930

**Published:** 2021-07-14

**Authors:** Shahin Nazar, Toine Pieters

**Affiliations:** ^1^Descartes Centre, Utrecht University, Utrecht, Netherlands; ^2^Freudenthal Institute, Utrecht University, Utrecht, Netherlands

**Keywords:** plandemic, disinformation campaign, Twitter, network analysis, conspiracy, public health

## Abstract

During the first few months of the COVID-19 pandemic, a 26-min documentary entitled *Plandemic* was released online and fanatically shared *via* Twitter and other major social media platforms. The producers of the documentary sought to spread misinformation and conspiracy theories and to discredit scientific experts using a sophisticated disinformation campaign. They set out to accomplish this by coaching citizens toward activism to maximize the speed at which the documentary propagated and decrease positive sentiments toward public health interventions. Applying techniques from social network analysis in conjunction with a formative content analysis of Twitter data, we examined the effectiveness of the *Plandemic* disinformation campaign as a case study of social engineering during the COVID-19 pandemic. By comparing the Twitter network's community structure and communication patterns before and after the release of the film, we demonstrate the *Plandemic* campaign to have been effective for two reasons. First, the campaign established a decentralized information sharing network on Twitter by coaching low-reach social media users to mass share the documentary, effectively subverting efforts to gatekeep its misinformation. Second, the campaign amplified negative sentiments regarding vaccination and containment measures among conspiracy theorists. These effects possibly have an indirect impact on the public's willingness to comply with public health measures. Our results show the necessity of further research about sophisticated social experiments such as the *Plandemic* disinformation campaign and provide important insights for policy-making to combat the spread of health misinformation during public health crises.

## Introduction

On May 4, 2020, a 26-min documentary entitled *Plandemic* was released online and fanatically shared *via* Twitter and other major social media platforms. The film's narrators argued that the COVID-19 virus was planned by global elites as a means of controlling the population. They further argued that vaccines are harmful and that wearing facial masks “activates” the coronavirus—meanwhile suggesting pseudoscientific treatment alternatives, such as “healing microbes” found in the ocean (1). Indeed, viewers of the documentary are faced with 26 min of Gish galloping, replete with misquotes from physicians and scientists and guest appearances from known conspiracy theorists. In essence, the documentary seeks to spread doubt, discredit scientific experts, and push people toward political extremes. In an effort to come to terms with the worlds of politics, economics, social movements, local/global relationships, events, and opinions and criticisms related to COVID-19, the film's arguments are presented in a seemingly objective way rivaling the sophistication of well-funded Hollywood movies based on “true stories.”

Health communicators and policy makers struggle to determine whether and how to intervene when a health topic becomes misdirected by a discourse characterized by falsehoods that are inconsistent with evidence-based medicine (2)^1^. This struggle becomes even more difficult when disinformation campaigns deploy strategies that increase receptivity to health misinformation. Mike Willis, the producer, narrator, and funder of *Plandemic*, uploaded the documentary on various social media platforms and *Plandemic*'s own website (4). A look at the website gives an idea of the film's sleek online marketing strategy as encompassed by, to name a few, email and scarcity marketing tactics. All of this is wrapped in a rhetoric fitting to what one might find in a revolutionary manifesto.

*Plandemic's* design as a piece of planned disinformation is unique in that the producer called for viewers and potential participants of the campaign to download and share the video themselves^2^. “In an effort to bypass the gatekeepers of free speech, we invite you to download this interview by simply clicking the button below, then uploading directly to all your favorite platforms” (6). Willis anticipated that the video would be banned from major social media platforms. In what can be seen as a high-caliber strategic public intervention akin to referral programs most commonly found in the marketer's toolkit, he used the *Plandemic* website to coach campaign participants in downloading and mass-sharing the film and thus, hopefully, bypassing media gatekeeping of any kind. The strategy also banked on the additional attention gained whenever someone sharing the documentary was banned or had their post-removed, adding to the hype cycle (7). It can therefore be said that by exploiting well-known marketing tactics and interventions for social change, Willis sought to maximize the reach, influence, and propagation speed of health disinformation and misleading claims.

Current research agendas on health disinformation and conspiracy theories are young and fragmented and emphasize causal explanations. It is well-established that a complex set of conditions and factors enable people to adopt beliefs in conspiracy theories, including personality types, heuristics, and cognitive biases to sociological factors such as level of education and political orientation (8, 9). Correlations between, for example, level of education and the likelihood of belief in conspiracy theories can be both positive and negative. Indeed, Chou et al. argue that “highly educated individuals may be equally vulnerable to misinformation when it comes to topics that are central to their identity.” Several political scientists and sociologists focused on consequences have found that conspiracy theories undermine public support for government policies, decrease key predictors of voluntary compliance, and can undermine health-related preventative behaviors (10–12). Van Prooijen et al. observe that “conspiracy theories originate particularly in crisis situations,” giving us reason to believe that the social consequences of health disinformation and resulting belief in conspiracy theories may be especially negative and severe during the COVID-19 pandemic. Examples include an unwillingness to receive vaccinations, rejection of conventional medicinal or dental treatments, or possible gravitation toward unsupported and potentially dangerous treatments based on pseudoscientific belief systems (13, 14). *Via* data-driven research, scholars have attempted to access and understand public sentiment and responses to the implementation of public health policies. Efforts to understand matters of public hesitance to vaccination or public perceptions of a pandemic are of great value to critical decision making in policy (15–17). However, these attempts at a better understanding of the nature of conspiracy theories have been replete with inquiries about the causality of beliefs while lacking research into consequences (18).

The focus of scholars investigating conspiracy theories has often been grounded in the assumption that conspiracy theories are “out there” as a set of circumstances, like a backdrop, without much regard for a conspiracy theory's etiological history (8). This focus may arise from a proclivity to think of different or overlapping conspiracy theories as interchangeable, centering on “typical” examples drawn from a limited assortment of contemporary favorites. At the same time, it has been acknowledged that disinformation campaigns are sponsored by specific actors, including governments, state-sponsored initiatives, private firms, or individual entrepreneurs as in the case of *Plandemic* (19). As can be seen, a lack of empirical research in how actors bring about misinformation limits scholarly engagement with the specific content and contexts of particular theories which might illustrate how they come about and gain meaning (8).

A second problem in this field of research concerns the replicability and ecological validity of conspiracy and disinformation studies. There is reason to believe that the literature's strong reliance on surveys may further hamper current research. The same can be said of researchers in political science who resort to experimental manipulation of attitudes in the pursuit to identify the variables and mechanisms involved in “conspiracist ideation.” Experiments and opinion surveys by nature lack a historical element and concentrate solely on cross-sectional studies. Indeed, little attention has been paid to how conspiracy theories and disinformation campaigns unfold over time, mainly because of the absence of meaningful time series data.

It should come as no surprise, then, that scholars of these topics take different stances on burgeoning theoretical problems. One relevant example is the current concern about how the internet and social media advances the spread and sophistication of disinformation (20). Some suggest that disinformation and conspiracy theories are indeed flourishing in the age of pervasive internet access, while others downplay its importance. Swire-Thompson and Lazer have applied these questions to the context of health disinformation, suggesting the need to further understand whether people are more or less misinformed by using the internet (21). Indeed, a growing number of studies evaluate the effect of social media and online disinformation campaigns on vaccination rates and attitudes (22). Yet, little research has been done into correlating the design goals of disinformation campaigns with their social consequences.

In *Understanding Conspiracy Theories, one* of the most comprehensive literature reviews to date, Douglas et al. (18) recommend inquiring into how conspiracy theories are communicated by actors and the social consequences thereof. The authors further suggest that one fruitful way of investigating consequences can be found in “the development of automatic coding of web content, of social networks in Web 2.0, and analyses of communication dynamics” [(18), p. 22]. These research avenues, they argue, open up “many opportunities to study the large-scale communication of conspiracy theories and its implications for social and political processes.” There have been a few studies along these lines of inquiry which have used Social Network Analysis. These studies have traced how conspiracy theories circulate through social networks and change the contents of discussions (23–27). Social Network Analysis can prove particularly effective in assessing how the network topology (defined as the specific patterns of connections between network actors) and social dynamics (patterns of interactions among the nodes over time and space) influence information disseminates in the given network (28, 29). Social Network Analysis therefore provides fertile terrain for investigating the effectiveness of the distribution strategies actors responsible for popularizing conspiracy theories employ.

Against this backdrop of methodological and theoretical gaps in the literature, we set out to investigate the effects of *Plandemic*'s release strategy on the network topology and social dynamics of the “plandemic” network on Twitter. Given that *Plandemic* was designed with specific intentions regarding a social network's topology and corresponding social dynamics, we asked: how did *Plandemic's* distribution strategy drive patterns of community activity and COVID-19 related communication on Twitter?

Our choice to use a Twitter retrieval methodology is justified as follows: by using Twitter data as the source of our analysis, we were able to access data on many conspiracy believers who might otherwise be difficult to reach (using survey or experimental studies). With this level of access, we aimed to bring much needed detail to the study of conspiracy theories and health misinformation— “in the wild” rather than in the lab. Using Twitter data also ameliorated burgeoning issues of replicability and ecological validity because the data and tools are publicly available. The quantitative tools of Social Network Analysis allowed us to process the effects of *Plandemic*'s release strategy in abstract, formal, and structural mapping of the network's topology. We used formative content analysis in conjunction with the Social Network Analysis to investigate how patterns of communication about COVID-19 related topics changed after the film's release.

Our findings are presented in three sections, as follows. First, in order to focus on the relationship between an important event— the release of *Plandemic*—and exposed actors, we characterized the effects of *Plandemic*'*s* release on the Twitter network's community structure and centrality properties. Second, we explored the effects of *Plandemic* on the particularities of information sharing: mainly what topics became more or less popular after the release of the film. In this second section, we also made inferences about the effects of *Plandemic* on user engagement with COVID-19 related notions and ideas and highlighted a few salient elements in their use of language. The final section is a discussion of the results in the context of our research question and concluding remarks about the implications of our analysis for health policy and further research.

## Materials and Methods

### Data Collection

The tweets in our collection of data were retrieved from George Washington University's publicly available dataset called *Tweetsets* (30). We filtered 188 million entries to only those tweets which contained the word “plandemic.” We determined a timeframe based on the following criteria: the timeframe should illustrate the growth and recession in popularity of “plandemic” tweets. Tweets mentioning “plandemic” for the first time spiked in the week leading up to the WHO's declaration of a global pandemic on March 11. It is therefore reasonable to take this first spike as the beginning. Moreover, popularity receded in the weeks after the release of the film. The final spike of popularity can be seen on the 4th of June as popularity receded in the following week. In the 4 weeks after the release of the film, popularity receded to a standstill. Therefore, in order to depict the “plandemic” discourse's increasing and receding levels of popularity we decided to include all tweets mentioning “plandemic” between March 3rd and June 10th, 2020. We retrieved a set of “dehydrated” (anonymized) tweets and “rehydrated” (de-anonymized) those tweets using Hydrator version 0.013 (31). The finalized dataset used for this study consists of 78,793 tweets from 42,966 users.

### Data Visualization and Analysis

Social Network Analysis focuses on the connections between actors and networks. It comprises a set of research methods used to model “relations and associations, developments and associations, and dynamic forces in networks and activities,” particularly on social media platforms [(32), p. 155]. The field's diverse range of applications has also proven useful beyond social and behavioral sciences, including for business and health informatics (33, 34). Based on a vast array of uses, the field's collection of theories, practices, and instruments is underscored by the following principles [(35), p. 205–222]: (1) the structure and characteristics of networks contribute to the performance of that system; (2) the relational state of actors impacts their behavior; and (3) the behavior of actors conforms to the network's (in our case, Twitter) environment.

We used Gephi, an open-source tool for social network visualization, to both quantitatively and visually analyze the structure and characteristics of the network within the study dataset (36). We did this first by defining the edges and vertices of our dataset. Edges, one of the two most central concepts in network theory, concern units of interaction, abstracted connections, or even physical immediacies (37). A relevant example of an edge (also referred to as a “link,” “tie,” or “relationship”) is the Twitter “mention.” According to Twitter's documentation, “a mention is a Tweet that contains another person's username anywhere in the body of the Tweet” (38). The other central concept in network theory is the notion of a “vertex” (37). Vertices (also called “agents,” “actors,” “nodes,” or “entities”) may be comprised of individuals, locations, events, or media. An edge accordingly links two vertices in any given social network. In our case, then, the ties between nodes in our network can be represented by who mentions who.

Visualizing any type of network means doing more than simply creating attractive images. The practice of constructing pictorial representations of networks for the use of evidence is nothing new and has persisted for centuries (39). According to Freeman (40), visualizations of social networks provide “new insights about network structure and have helped communicate those insights to others” (p. 1). Examples of “new insights” may include accurate measures of the impact of scientific research, disaster responses in communities, or policy diffusion.

In order to visualize the network's topology and therefore better understand the information dynamics of the network, we ran the OpenOrd, ForceAtlas2, and Yifan Hu Multilevel layout algorithms (in order of mention) (41–43). These layout algorithms are useful for decreasing visual complexity and improving intelligibility of a network's characteristics, especially when interpreted in conjunction with graphs. After applying the appropriate layout algorithms, we calculated relevant network metrics to color the graphs according to the outputs. For the purpose of this research, the following three metrics were calculated: modularity, in- and out-degree, and betweenness-centrality.

Modularity is a measure indicating the level of sophistication of a network's internal structure. An internal structure, referred to as the community structure, describes the degree to which a network is compartmentalized into sub-networks. The modularity's capacity allows us to depict the degree to which information sharing is divided, unified, fragmented, or clustered within the network. Modularity as a measure was especially useful in answering our research question because we were able to benchmark the degree to which *Plandemic*'s distribution strategy was effective at decentralizing information sharing by calling individuals to engage in “lone-actor” behavior. We used the community detection algorithm developed by Blondel et al. due to its accuracy and accessibility (44).

Centrality is a measure of the importance of any given node in the Twitter network (45). Being that the *Plandemic* network is directed (i.e., that an edge has a one-way orientation), we calculated both in-degree and out-degree centrality. The in-degree centrality of a node concerns the number of other nodes that point toward it. We can regard in-degree centrality, then, as a measure of popularity and influence, allowing us to find prominent users in the dataset. Correspondingly, out-degree centrality refers to the number of edges that point away from a node. A Twitter user with a high out-degree measure can be referred to as a highly active node. Locating the central nodes gave insight into how *Plandemic*'s release strategy affected the most important or active nodes in the network (46).

Betweenness, the third measure, is also a centrality measure. Technically, betweenness specifies how often a given node appears on the shortest paths between other nodes in the network (47). What this means is that a Twitter user with the highest betweenness centrality can be seen as a bridge in the network. Such Twitter users are important in the overall network because they carry a significant portion of the information flow. Network researchers have introduced several betweenness measures and deciding between any one of these measures brings to the fore distinct benefits and shortcomings. It can be said that there exist two classes of betweenness measures relevant to our case study: random-walk betweenness and shortest-path betweenness. Newman stressed how deciding between the two algorithms depends on the directionality of the information flow within a network. Random-walk betweenness best “represents information that has no idea where it is going” whereas shortest-path measures like Brandes' algorithm best represent “information that knows precisely where it is going” [(48), p. 42]. The *Plandemic* case study involves a directed network because a mention is best represented as a one way relationship between two nodes. Given then that we are dealing with an information flow “that knows precisely where it is going,” we may regard the shortest-path measure as the most reasonable choice out of two. Our choice of measure does not, however, invalidate the usefulness of random-walk betweenness. According to Newman, it may rather “be of use to compare the predictions of the two measures to see how and by how much they differ” [(48), p. 42]. Thus, other measures may aid in obtaining a more comprehensive understanding of the *Plandemic* documentary and its discourse. Given the aforementioned evaluations, we used Brandes' betweenness centrality algorithm (49), which allowed us to calculate the average path length in both networks and in effect provide insights into how efficiently information is disseminated in the network. By correlating our betweenness measure with the abovementioned measures, it becomes possible to provide a robust analysis of *Plandemic*'s effect on the Twitter network's information dissemination capabilities and, therefore, how people share information on a particular social media platform.

### Content Analysis

Purely structural and statistical changes in social networks can provide limited explanations of changes in priorities and engagement. Indeed, structural changes say little about the “life worlds” of individuals pulled into activism by clever messaging and tactics (50). We must remember that the aim of this research is to understand how planned disinformation affects social media network dynamics and public engagement with COVID-19 related science and technology. The term *social dynamics* in general refers to the interactions, relationships, peer/networks/structures, interpersonal roles, cultures, and norms which actors co-construct to organize social contexts (51). Hence, these dynamics are relational, negotiable, and fluid, just like a social network on Twitter. Fortunately for the researcher, this complexity is somewhat reduced on social media platforms where units of engagement are predefined: users can only post up to 150 characters, algorithms filter what information they see, buttons allow a certain range of symbolic interactions, UI design nudges users toward certain behaviors, and so on. It is therefore both fruitful and economical to investigate how *Plandemic* affected engagement on Twitter to come to a more robust answer to the research question.

For this reason, the second part of our study consisted of a formative content analysis. Using NVIVO 11, we first queried the most frequently occurring words and determined a predefined list of topic categorizations based on the query results. Then, using the open source Python (version 3.8.8) library “Pandas” version 1.2.3 along with Python's statistics module, we labeled the Tweets with their respective topics and exported the dataset as a.CSV file (52, 53). A subsequent statistical analysis was conducted using Pandas, and Office 2016's Excel (including Toolpak) in a second round of general calculations and data clean-up in order to verify the output by Pandas (54). Based on this statistical analysis of labeled data, it was possible to determine which elements of discourse changed due to the film's release. In addition, we correlated tweet attributes with the release of the film and evaluated the differences between pre- and post-film in terms of proxy measures, mostly for reach and popularity. Here, reach and popularity can be viewed as dependent variables and were invoked by the number of retweets and likes—both of which serve as measures of a tweet's potential to make impressions on Twitter followers and even other social networks.

## Results

**Figure 1:**
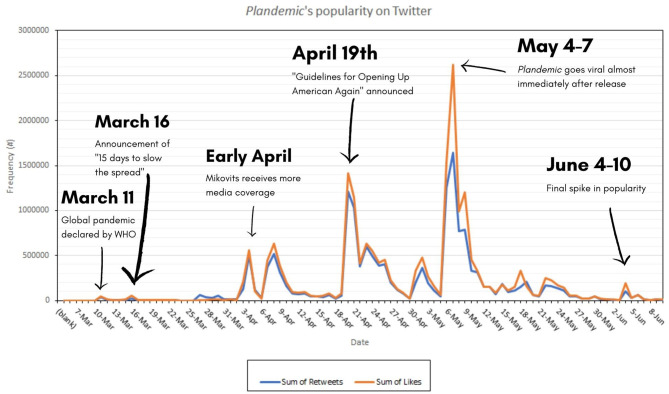
Volume of tweets using #Plandemic over time, data from 2020. Key events that received significant publicity were added.

Figure 1 is a graph that illustrates the video's spread using the sum of all interactions in the dataset. We can see the escalation very clearly. The film caused a surge of new tweets, twice as many tweets in the 2 weeks following the documentary's release compared to the previous 2 months. It is quite evident from this data that *Plandemic* indeed obtained “viral” fame. The dataset shows a tweet count of 30,368 in the period leading up to the film's release. Around 66% (*n* = 20,080) were retweets and 6% (*n* = 1,954) were replies. The dataset contains a tweet count of 48,425 in the period after the film's release. Around 65% (*n* = 31,535) were retweets and 6% (*n* = 3,057) were replies.

**Figure 2:**
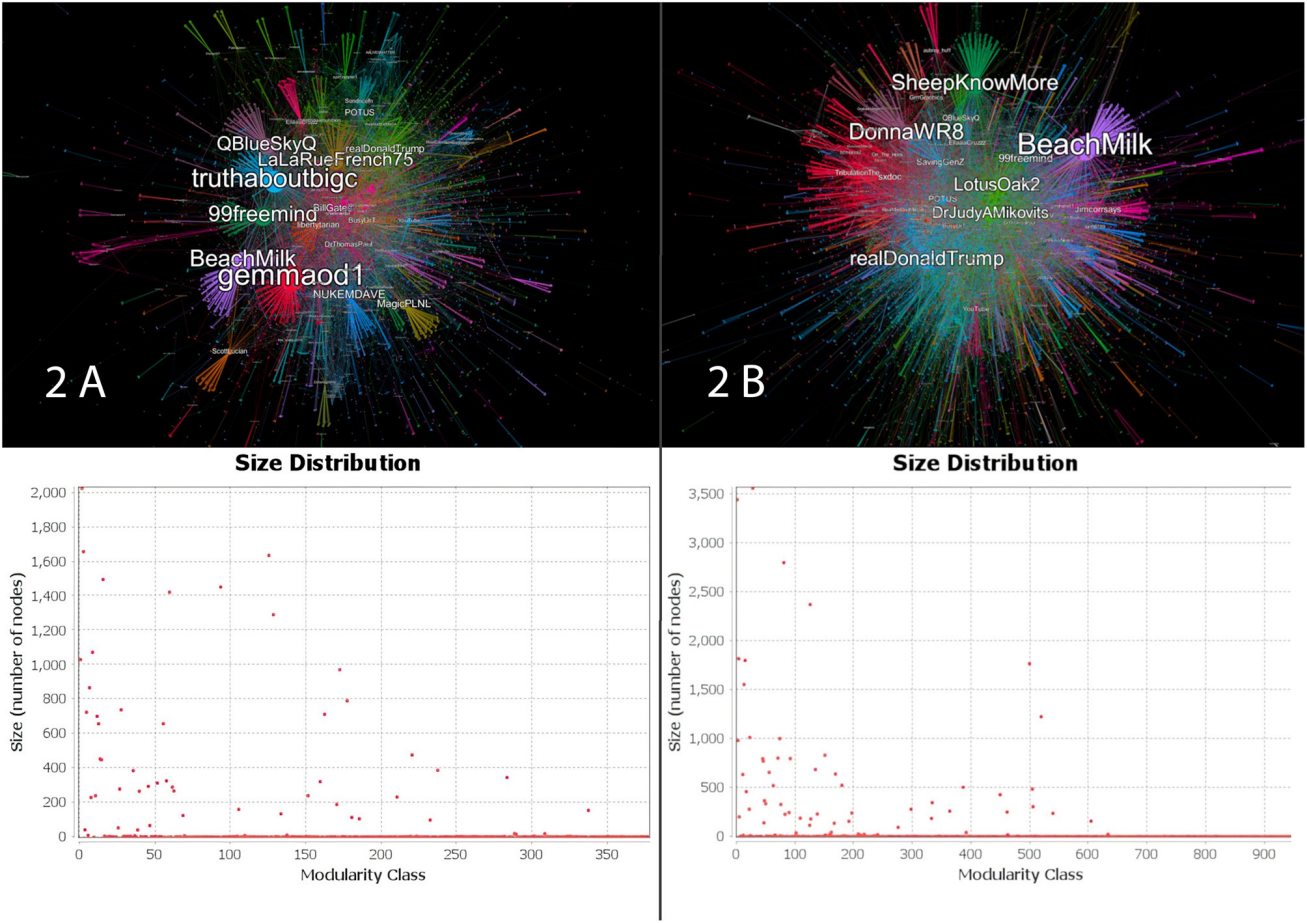
In **(A)** we see the pre-release network and graph and in **(B)** the post-release network. Each community is assigned a color. The spatially closer different communities are the more users these communities share as “bridges” between them. The size of a user is likewise directly proportionate to its popularity within its community and over the entire network—as determined by the node's in-degree. Correspondingly, the two size distributions below their corresponding networks are a quantitative representation of the two visualizations. For the pre-release network, the algorithm's output was a modularity of 0.768 with a total of 378 communities detected. The post-release network resulted in a modularity of 0.773, with a total of 972 communities detected—roughly three times more than pre-release.

*Plandemic*'s surging popularity also had a significant effect on the network's internal structure. In Figure 2A, we can see that communities were larger and more compartmentalized before the release of the film. After the film's release, as seen in Figure 2B, colors fade in and out of one another in a blurrier smudge of overlapping communities. Most communities also became much smaller. These changes in the network's internal structure suggest that after the release of the film, low-reach Twitter users engaged more often with each other, bypassing the large nodes.

However, modularity is not the only indicator of how information disseminates in social media networks. This pattern of modularity change may still be attributed to bursts of popularity, as shown in Figure 1—characterized by many people suddenly posting *Plandemic*-related content as part of a hype-cycle.

**Figure 3:**
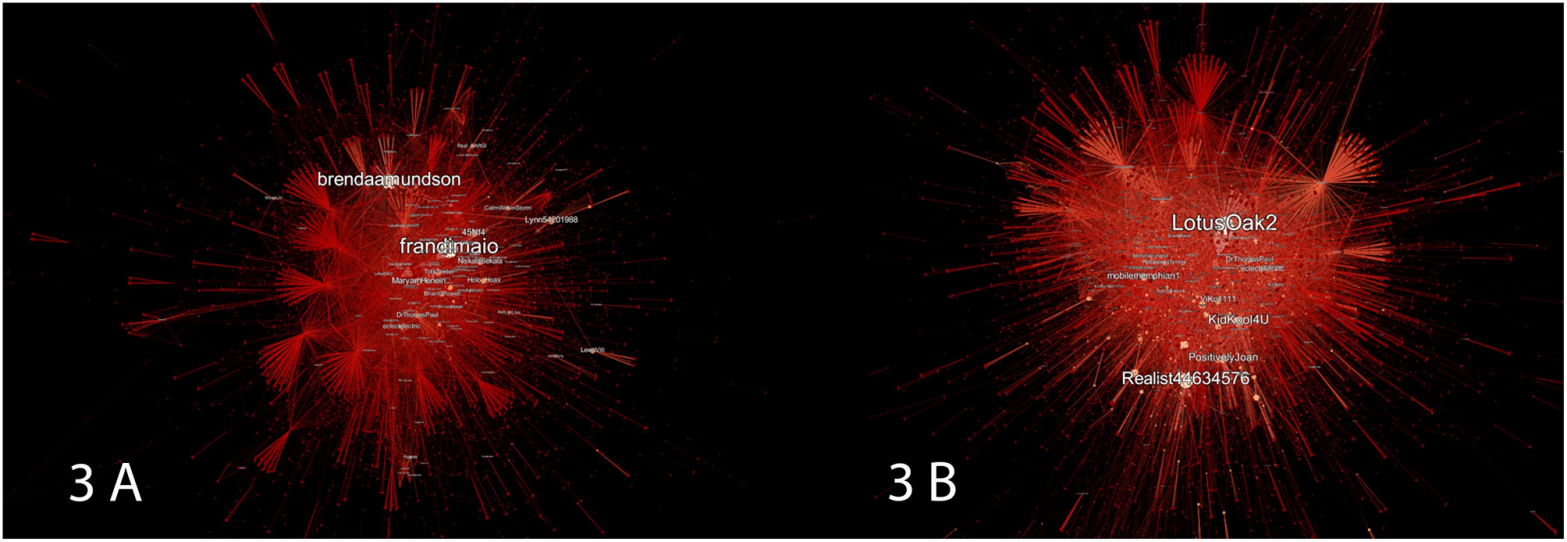
In **(A)** we see the pre-release network and in **(B)** the post-release network. The nodes and edges of the pre- and post-release networks are colored according to their average out-degree measure. Likewise, the size of any given node corresponds to its average out-degree. Deeper red areas indicate lower out-degrees of nodes, and the lighter-red a node or edge is the higher its corresponding out-degree.

The out-degree centrality of the network's nodes is depicted in Figure 3 and can illustrate how the film's release contributed to the network's information sharing capacities. We can see that the pre-release network is comprised of much darker reds, whereas the opposite characteristics can be seen in the post-release network. We can accordingly correlate these results with Figures 2A,B output and infer that the post-release network not only consisted of a high number of small communities, but that their members were also more consistently active and vocal than before.

**Figure 4:**
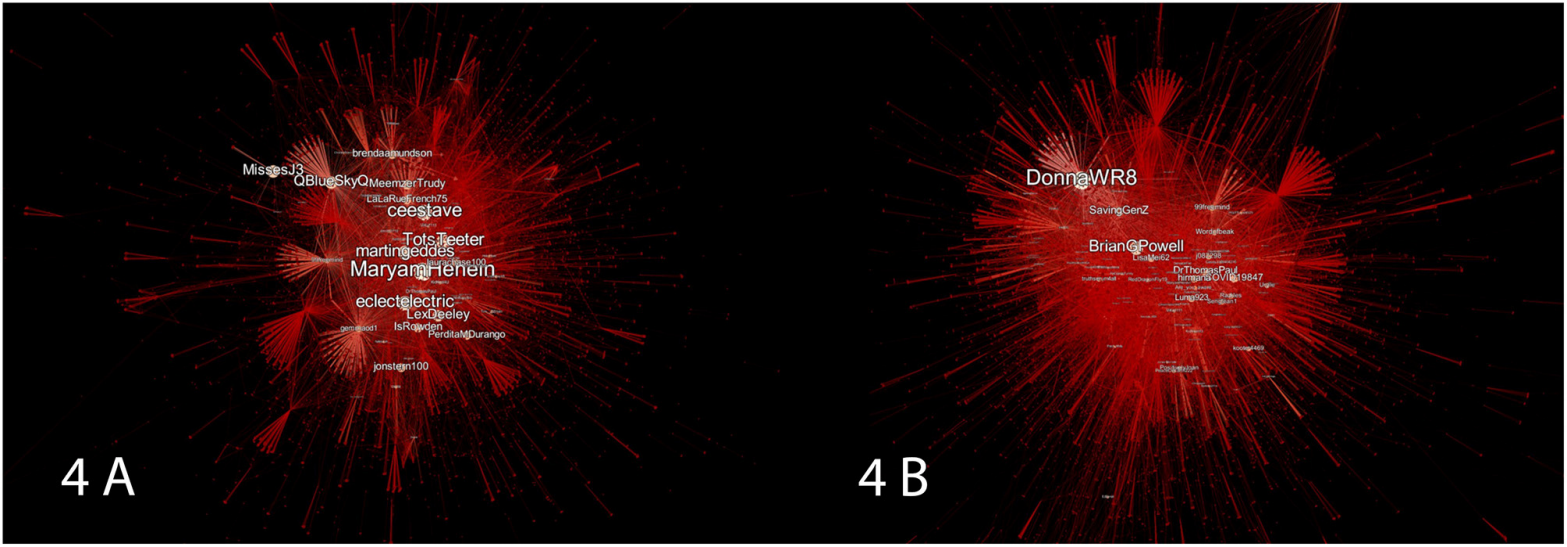
In **(A)** we see the pre-release network and in **(B)** the post-release network. The nodes and edge of the pre- and post-release networks are colored according to their betweenness-centrality. The size of any given node corresponds to its betweenness-centrality measure. Deeper red nodes and edges indicate a lower betweenness-centrality score, while lighter-red counterparts correspond to a higher measure of betweenness-centrality.

Figure 4 represents the changes in betweenness-centrality of the pre- and post-release network, which can be described both visually and quantitatively. Visually, we can see a strip of lighter nodes going through both networks. We may imagine these two strips acting as the central bridges in the network, like the backbone that does the heavy lifting when it comes to getting information around the network. Because of its dense strip of lighter nodes, the pre-release network appears much more dependent on central hubs or bridges for disseminating information across the network. The post-release network is much less dependent. Its prevalence of dark red hues and a sparser strip of lighter nodes indicates that more information is passing through the network *via* nodes which have less connection to other groups in the graph. Quantitatively speaking, the average path length of the pre-release network is 10.38 with a diameter of 25, while for the post-release network the average path length is 8.91 with a diameter of 29. Although the post-release network had, on average, shorter paths (therefore allowing for more efficient distribution of information), there were also less gatekeepers present to control the dissemination of information. Not only, then, is the post-release network more efficient, but information is propagated in a more decentralized manner than in the pre-release network.

While we have reason to infer that *Plandemic*'s call to citizen activism was effective, we know little about how changes in user engagement may be attributed to *Plandemic*'s call to activism. How, we may then ask, did *Plandemic*'s marketing and distribution strategy change the community's engagement and priorities regarding COVID-19?

**Figure 5:**
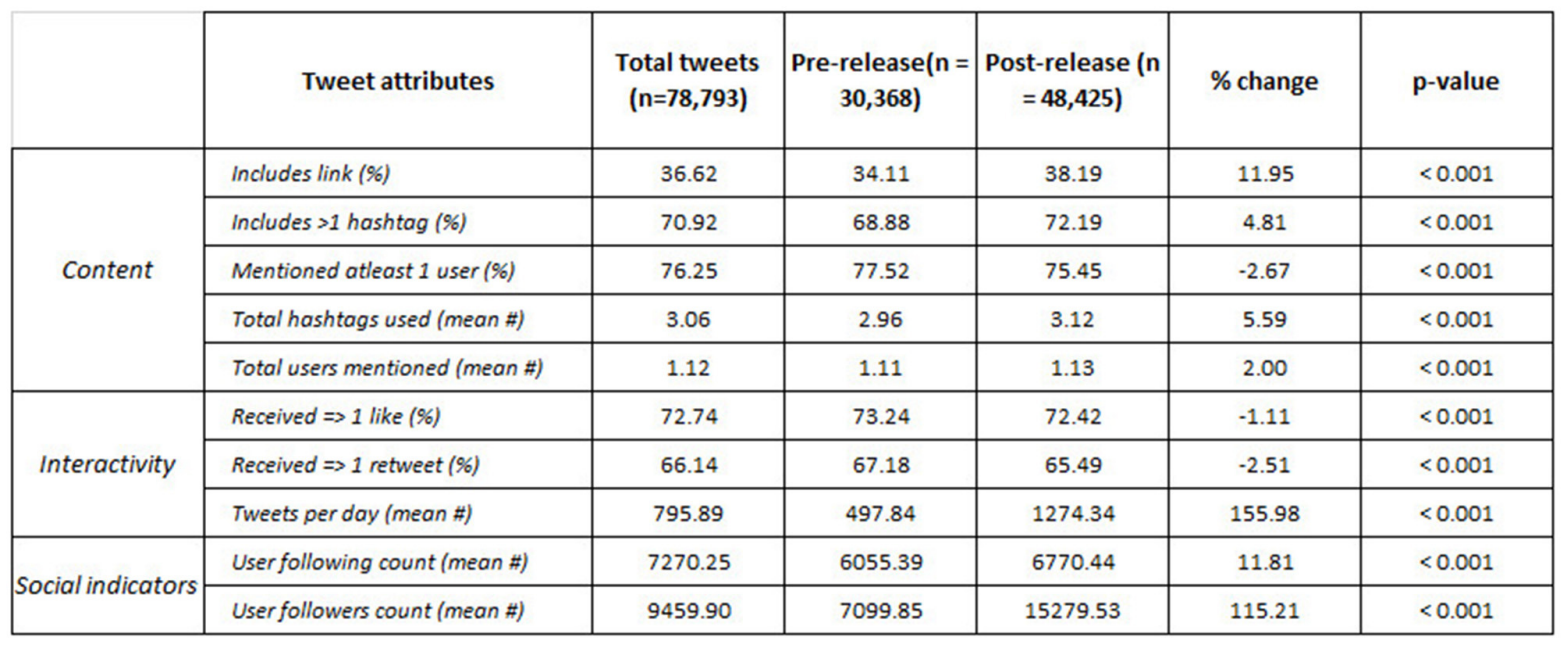
Differences pre- vs. post-release. All tweets (*n* = 78,794) were collected between March 3 and June 10, 2020. Characteristics are stratified by pre- vs. post-film periods and sum across rows. Percent change between pre- vs. post-film release is presented in the “% change” row. Tweet characteristics are not mutually exclusive. The first three columns are in absolute number units. Chi-squared and Welch's *t*-test are used to assess the difference pre- against post-release (alpha = 0.05).

An average of 796 tweets per day contained the term “Plandemic.” The average number of tweets per day increased by more than 155 percent (from around 498 tweets pre-film to more than 1,274 post-film, *p* ≤ 0.001). Significant increases in activity also corresponded with the average number of followers per user in the network more than doubling (115%, *p* ≤ 0.001) after the film's release, as can be seen in Figure 5. The average number tweets that received more than one like and one retweet did not change significantly (1%, *p* ≤ 0.001, and 2.5%, *p* ≤ 0.001, respectively), suggesting that overall user engagement remained the same despite a much larger volume of activity occurring between low-reach users after the film's release.

**Figure 6:**
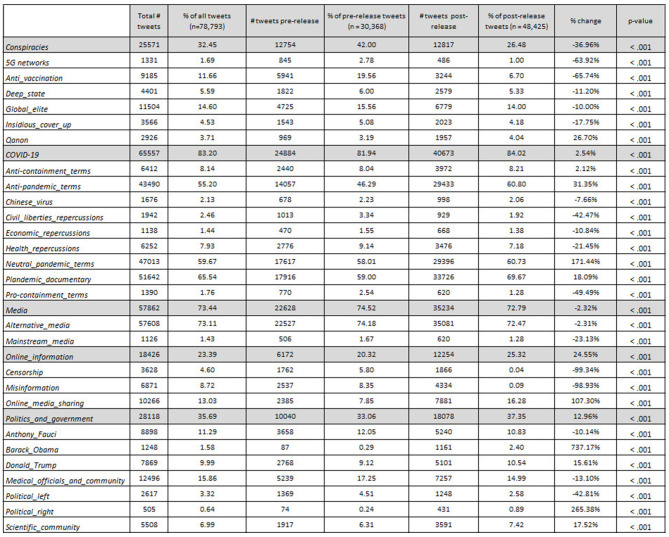
Percentages of most prominent topics mentioned in the dataset. Topics were coded according to a word frequency query (top 2,000 words with at least 3 letters) in NVIVO. Percent change between pre- and post-film presented in the rightmost column. Chi-squared and Welch's *t*-test are used to assess the difference pre- against post-release (alpha = 0.05).

**Figure 7:**
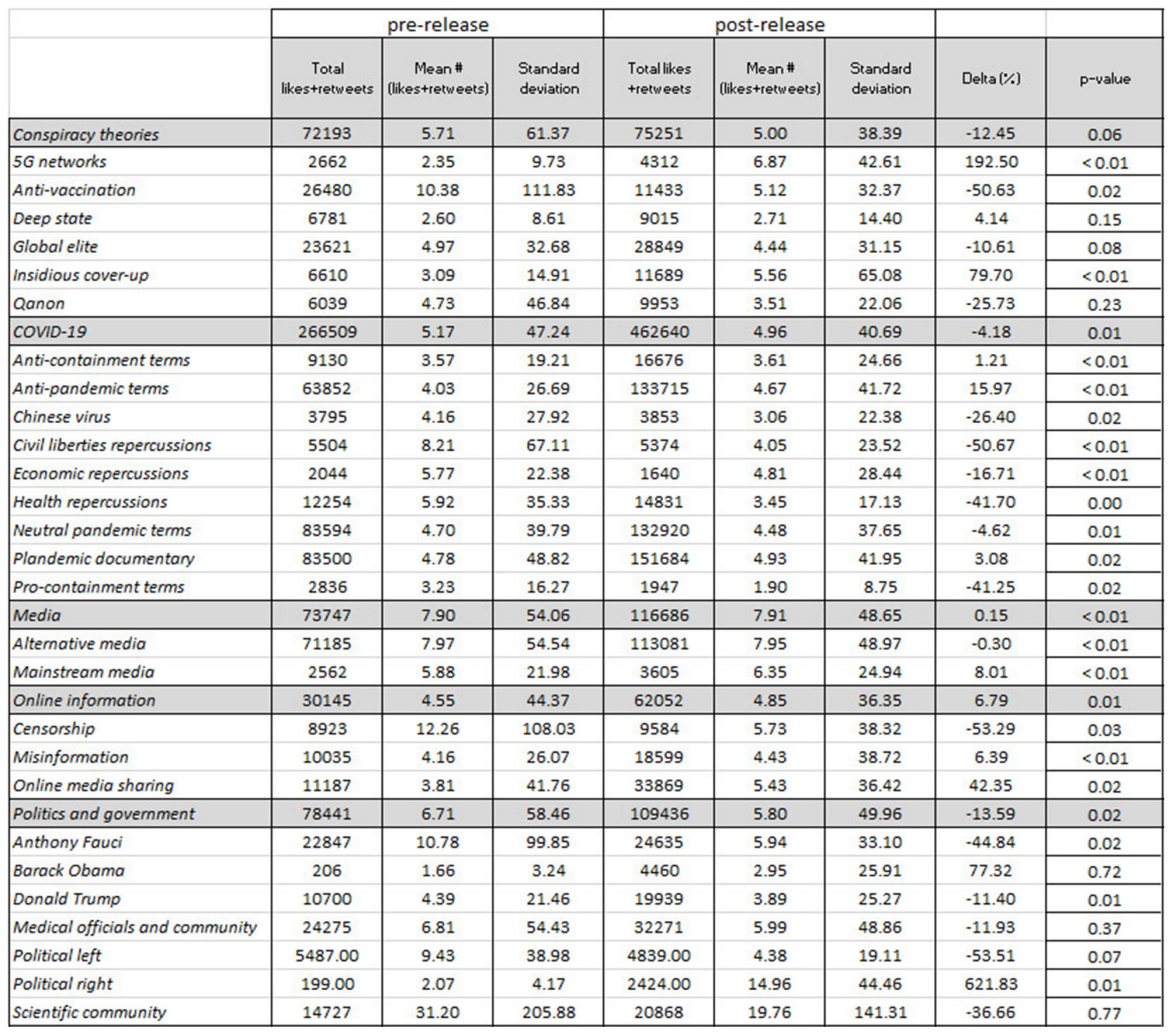
Average likes + retweets for “plandemic” tweets by topic category. All tweets (*n* = 78,794) were collected between March 3 and June 10, 2020. Percent change is presented in the final column. Chi-squared and Welch's *t*-test are used to assess the difference pre- against post-release (alpha = 0.05).

In Figure 6, there are three distinct patterns of interest concerning information sharing and media, conspiracies, and politics and government. First, online *Plandemic* media sharing (in particular non-YouTube media) flourished, with an increase of about 107%, *p* ≤ 0.001, after May 4, 2020. In Figure 7, we see that tweets that shared online media not only increased in proportion to total tweets but also received 40%, *p* = 0.02, more engagement. This may be due to platforms like YouTube restricting access to the film (and at times banning it altogether) in tandem with the producer's call for campaign participants to share the film *via* alternative video sharing platforms. We can see in Figure 6 that at least 16 percent of all tweets were sharing media. Online media sharing ranks among the highest mentioned topics in proportion to total tweets, second only to more general topics and alternative news mentions. Interestingly, <1% of tweets mentioned false information or censorship after the release of the film, with both topics decreasing by about 100%, *p* ≤ 0.001, in comparison to before. Tweets with keywords about censorship also received 50%, *p* = 0.03, less engagement post-release, as can be seen in Figure 7. Lastly, more than 70% of all tweets mentioned alternative media sources (e.g., Rush Limbaugh, David Icke, RT news, Sean Hannity, and Graham Ledger).

About 40% of total pre-release tweets discussed a subgenre of conspiracy theories, which decreased after the release of the film to about a quarter of total tweets discussing a subgenre. As we can see in Figure 6, the most common conspiracy theories listed were about anti-vaccination and the “global elite” (more specifically embodied by Bill Gates). One point of interest is the fact that after the release of the film, almost every conspiracy theory became less prominent in terms of volume, with the exception of QAnon which occupied the discourse of more than 25% of total post-release tweets. Discussions about 5G networks and anti-vaccination decreased the most (~60%, *p* ≤ 0.001) after the release of *Plandemic*. Nevertheless, in Figure 7 we can see that after the release of the film, conspiracy theories about 5G networks and insidious cover-ups still fared well, with a significant increase in engagement (~190%, *p* ≤ 0.01 and 80%, *p* ≤ 0.01, respectively). This indicates that while there were fewer tweets about the 5G network conspiracy theory, the tweets that were produced received higher engagement in terms of likes and retweets than those published before the release of the film. Along similar lines, we can see that pro-containment terms also dropped significantly, indicating that the film managed to diminish favorable views of containment at least temporarily as a means of combating the pandemic.

Lastly, the film successfully drew attention to specific public figures. The three most frequently mentioned public figures were US president Donald Trump, Dr. Anthony Fauci, and Barack Obama. Donald Trump and Anthony Fauci dominated discourse after the release of the film, with both figures occupying at least 10% of all tweets (despite Fauci being mentioned about 10% less than before the release). Similarly, Barack Obama was mentioned at least 700%, *p* ≤ 0.001, more often post-release than pre-release. As can be seen in Figure 7, *p* = 0.01, tweets about the political right gained 620% more engagement, significantly outnumbering any other topic.

**Figure 8:**
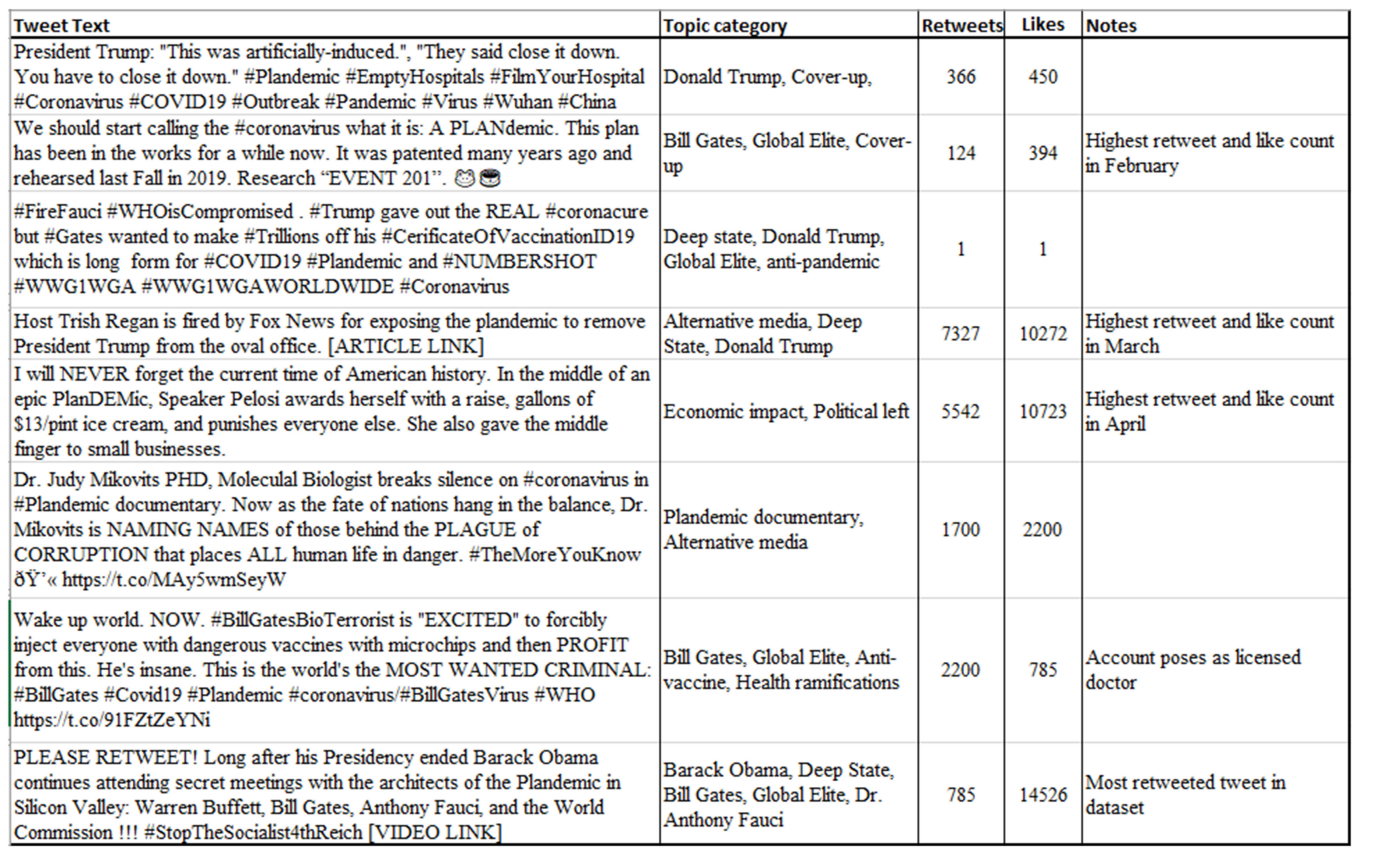
Examples of early and popular tweets mentioning “plandemic.” Qualitative descriptions are provided for additional context in the second column from the left. All tweets are from March 3 to June 10, 2020. For privacy reasons, all tweets are organized in ascending order by date without mention of specific date, user, or link. Retweets and likes may not represent current values, as the dataset was retrieved before publishing this paper.

In Figure 8, we provide example tweets to offer a general idea of the rhetoric seen in the dataset. One point of interest is how the overall tone of the tweets encourages other like-minded people to do their own “research” as an act of public or civic participation, whether *via* field research (#FilmYourHospital) or a call to research “EVENT 201.” Overall, it appears that *Plandemic*'s distribution strategy further engrained the mindset of conspiracy thinkers to take matters into their own hands.

Yet there are no tangible indicators of how increased activism and participation served to develop these conspiracy theories into more robust or fleshed out iterations. Rather, it appears that while Twitter enabled this discourse to begin with, it likewise may have limited the discourse's development in terms of conspiracy theories. At first glance, we see what one might expect of conspiracist discourse dominated by statements <280 characters: Gish galloping, statements containing mixtures of fact and fiction that tend to lend credibility to the latter, and so on. In general, it appears that conspiracist tweeters turn their attention less to news content and more to the positioning of an issue.

**Figure 9:**
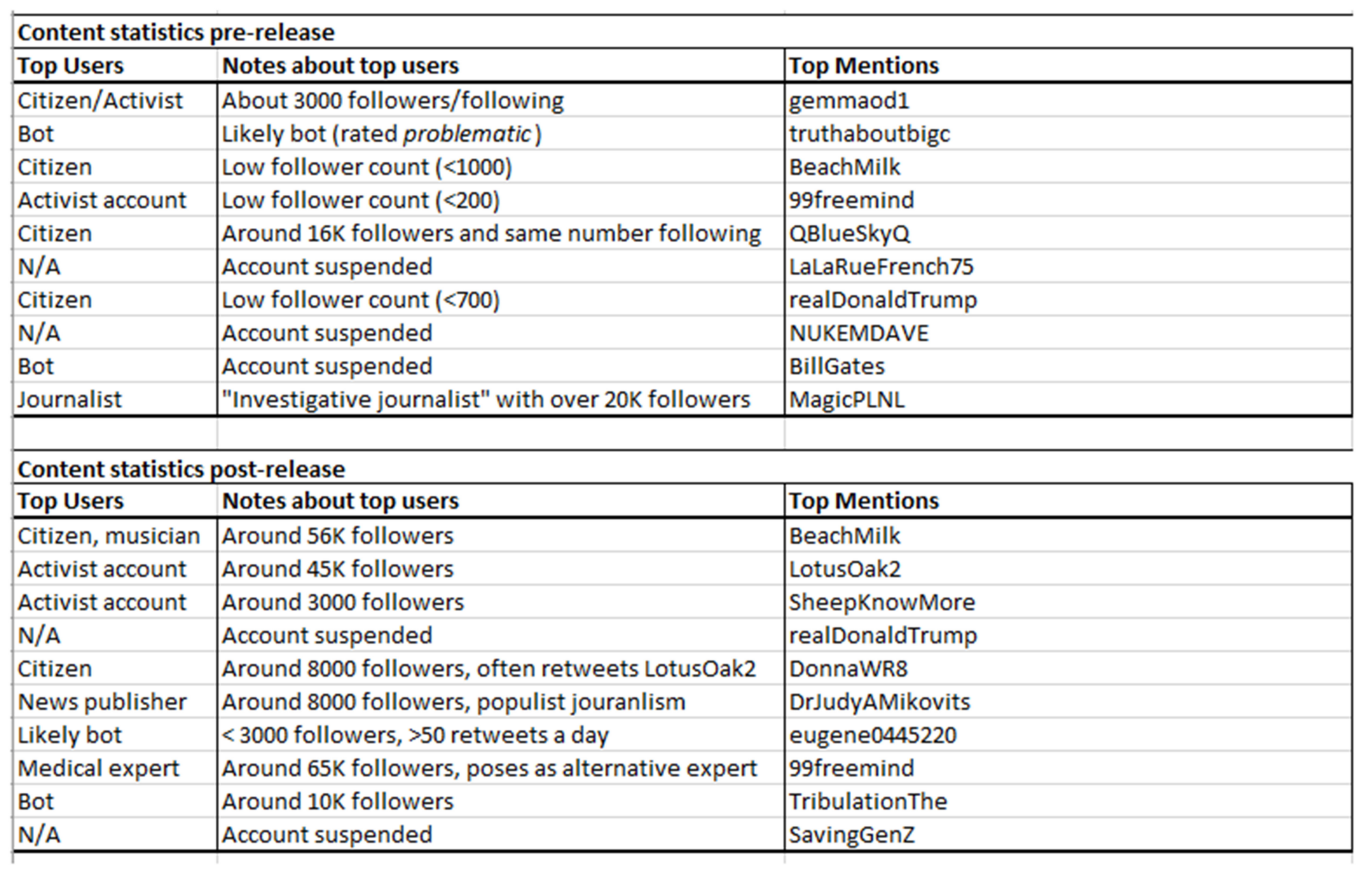
This table depicts the most influential users and provides a short description of the user account. The first column provides an outline for the type of account, and the second column provides more general information. The names of the accounts are redacted for privacy reasons. Every account was screened through BotSentinel, a webapp that checks for unusual social media behavior, and those with a score of 80% or above were manually marked as bots. The follower count is based on the number of followers the users had during the period of data retrieval.

Likewise, the most influential Twitter users depicted in Figure 9 appear to be either citizens or activist accounts, rather than bots, and the most common word in more than half of all top users' profile descriptions was “truth.” These profile descriptions often signal a search for a “hidden truth,” as if they are part of a citizen initiative to purge the world of evil actors. It appears that *Plandemic* and other conspiracy theories such as QAnon, that hitched a ride on *Plandemic*'s popularity (or vice versa), made Twitter users feel as if they were working together in an act of community participation under a sense of community victimization.

It is especially the idea that hidden truths are revealed in subtle symbolism and innuendo's that comes to the fore in the dataset, as it is replete with suggestive wordplays and words taken out of context—such as spelling “plandemic” and “planDEMic,” implying democrats as the masterminds behind the “plandemic.” Along similar lines, experts are often quoted out of context—for example, how #BillGatesBioTerrorist is “EXCITED” to forcibly inject people, implying that Bill Gates reveals inklings of evil intentions in his use of wording—in attempts to reveal hidden innuendos and readings between the lines of expert testimonies. We can see that this form of overt hypervigilance leads to the generation of conjectures and hypotheses which can be distinguished by their reliance on finding patterns, connections, or hidden meanings in what might otherwise be causally unrelated events.

## Discussion

Our aim in this article has been to bring to light the consequences of *Plandemic*'s release strategy on information sharing and the social dynamics of Twitter networks. We observed that almost immediately after the WHO declared a worldwide pandemic, *Plandemic* became a frequently used social media term and hashtag associated with an assemblage of popular conspiracy theories. The term “plandemic” was already frequently used on social media before the documentary's release. It appears that *Plandemic* leveraged pre-existing beliefs about the nature of COVID-19, as seen in the way that the film generated repeated bursts of Twitter activity over the course of a few months.

As we have seen, the documentary's release strategy was deliberately designed to amplify the distribution of *Plandemic*. One important element of the overall strategy entailed instructing individual social media users to actively participate in distributing copies of the film. The results of our network analysis were consistent with the aims of this public intervention, indicating that the campaign was a success insofar as the disinformation network indeed became more decentralized, relying on a densely connected network of small communities that consisted of users who had limited reach but high output (especially when it came to online media sharing). It is evident that *Plandemic* instilled a kind of *ad hoc* coordination between citizen Twitter users. This effect is confirmed by content and user analysis, where we found that users shared more media and actively encouraged others to participate in research and unveil COVID-19 related “truths” which directly opposed public health measures.

Research on the epistemic values prevalent in online communities of people who are swayed by disinformation campaigns might therefore provide fruitful avenues to better understand how disinformation campaigns shape the contours of beliefs systems. Epistemological terms common in how Twitter users express their opinions, such as “truth” and “research,” should therefore be the focus of future inquiries about the epistemic values and virtues prevalent in the post-truth era.

We have shown with publicly available data that social disinformation experiments have significant consequences for what and how the public talks about public health policies and shares information regarding public health matters. Anti-vaccination content, while not increasing in popularity, gained a burst in engagement (with fewer people tweeting about it but published tweets receiving significantly more likes and retweets). *Plandemic* was therefore successful in amplifying medically unjustified fears of vaccination. The sharp drop in pro-containment terms also indicates that *Plandemic* was successful in at least temporarily diminishing people's willingness to comply with public containment measures. It can be said that interventions like *Plandemic* and its consequences have a clear impact both on network dynamics and social media, but in addition its campaign had consequences for perceptions and beliefs regarding vaccination and isolation (social distancing and containment). The question remains as to what extent *Plandemic* affected sentiments in the general population. Nevertheless, its significant impact on conspiracy theorists shows that it is fruitful to investigate the broader impacts of the film as conspiracy theorists are not an isolated demographic group. The indirect effects of disinformation campaigns may therefore be equally important to understand than the direct effects.

The possible implications for policy and research will be pointed out. Suppose *Plandemic* had been released right before January 2021, or maybe a day or a week before a major public vaccination? We can now show that disinformation spread would be much harder to gatekeep due to the decentralized nature of information diffusion online, and that, consequently, a significant proportion of the public might resist public health policies. Policy makers, public health officials, and medical information gatekeepers do not yet have the appropriate tools at their disposal to combat these disinformation campaigns, as was the case with *Plandemic*. Now that we have established that public interventions like *Plandemic* are akin to social experiments, being that they have clear methods and goals in mind, it would be useful to further investigate their consequences on network dynamics and social media, especially regarding how they advance the actual content and argumentative strength of conspiracy theories. Case studies of social experiments like *Plandemic*, taken as a more concrete backdrop of the broader phenomenon of disinformation campaigns, provide insight into who creates disinformation, how, with what idiosyncratic methods. Thus, advancing research in this direction will allow for more pro-active, tailored, and prolonged efforts to combat the ever-evolving strategies employed to sow disinformation.

Using Social Network Analysis on Twitter data has proven especially useful for policy makers. First, social network visualizations can show the direct impact of public interventions like *Plandemic* on the dissemination of disinformation. It is then possible to support ongoing monitoring or surveillance of social media platforms using robust, up-to-date systems for tracking the emergence of misinformation and conspiracy theories. Further research on how social experiments like *Plandemic* affect social relations can be fruitful for supporting policy makers in a wide range of scenarios. Do other social experiments like *Plandemic* incur similar changes in community structures, or do different network topologies correspond to different intervention strategies employed by disinformation campaigns? Is it possible, by investigating additional cases of social experiments which resemble that of *Plandemic*, to come to a classification of different types of network outcomes so that policy makers might respond to patterns of social organization in real time? We suggest that these are important questions to answer in future research endeavors. Given that social media is rife with ever-changing and sophisticated tactics of spreading disinformation, policy making needs to be able to efficiently address ongoing bursts of disinformation, particularly during times of public health crisis.

## Strengths and Limitations

At its core, the methods and results of this study reinforce the usefulness of previous mixed-method work by, for instance, Bruns et al. (25). The authors draw from a mixture of quantitative and qualitative methods, including network analysis, to illustrate the development dynamics of a simple rumor that turned into a widely-endorsed conspiracy theory. Similar to the current study at hand, they visualize the Facebook network structures to confirm evaluations made with another method: a time-series analysis that is at the core of the article. Pascual et al. use social analysis in their study as a way to “provide a bird's eye view of public discourse online” [(55), p. 2]. The same can be said about how Haupt. et al. analyze Twitter discourse related to the “Liberate” Protest movement by way of, among other innovative techniques, an analysis of topic clusters and structural characteristics of retweet networks (56). All in all, scholars employing a mixed-method social network analysis tend to acknowledge the interpretative complexities that are distinct to online social movements and tend to use social network analysis as a way to further contextualize other forms of (qualitative) data.

Our findings contribute to a growing literature on the challenges of how citizen movements may be coordinated to further the spread of misinformation during COVID-19. Results by Ahmed et al. and Haupt et al. confirm that different forms of citizen participation should come to the fore as objects of analysis as such, mainly due to how citizen accounts tend to be effective communicators and opposers to public health measures tend to be better organized than their counterparts (56, 57). We compliment these works by showing how citizen movements can be altered by calls to action promoted by marketing and distribution campaigns, with the capacity to enhance individual participation and collective coordination with conspiracy theory narratives.

Our results suggest that disinformation campaigns may deliberately fragment discourse as to withhold traditional gatekeeping techniques to be performed adequately. Pascual-Ferrá et al. discern that “fragmented discourse, especially when it distracts or misguides the public from what they must do to protect themselves and others, has the potential to worsen the impact of a pandemic” [(55), p. 2]. Prior studies have found that ameliorating the effects of citizen-based movements by counteracting misinformation with “untargeted, trustworthy information, delivered from public health authorities as well as popular culture influencers” [(57), p. 6]. We anticipate that the findings of this study will provide a useful point of departure of future work that aims to identify how to further refine recommendations to counter disinformation tactics. Fortunately, this can also be tested with the already released Plandemic sequel and subsequent releases because the campaign is still running.

According to Haupt et al., “previous social media research shows that while a majority of user behavior is passive and mostly involves simply browsing through content, hyperactive users on social media have agenda-setting effects” on the respective discourse and the shaping of public opinion [(56), p. 2]. Ahmed et al. (57) observed this tendency in their study about the #FilmYourHospital movement. The authors found that major disseminators of the #FilmYourHospital conspiracy were in fact popular citizen accounts [(57), p. 2]. On the other hand, Gruzd and Mai found out about the #FilmYourHospital movement that “much of the content came from users with limited reach” with just a “handful of prominent conservative politicians and far-right activists” serving as the initial boost to kickstart the hype surrounding #FilmYourHospital [(26), p. 7]. Given the current study, we emphasize that accounts with low-reach can indeed carry a significant part of the information sharing on a network. Indeed, in similar ways as Gruzd and Mai observe “opportunistic political activists […] exploit conspiracy theories during COVID-19,” we view the producers of *Plandemic* as actors with similar exploitative tendencies. It is important to note that the different results between these various studies come to the fore as a result of picking different actors as our points of focus. As a result, different results should not be seen mutually exclusive and together depict a more nuanced view of citizen activism during COVID-19.

One limitation of our methodology pertains to the extent to which the current findings are generalizable to similar conspiracy theories on, for instance, other social media platforms, because the data used in this study comes solely from the Twitter API. As Marres and Moats pointed out, “the factors analyzed are those that happen to be reported by,” in our case, Twitter [(58), p. 5]. This issue of whether we are investigating platform-specific dynamics or patterns generalizable to other facets of the digital social world remains an ongoing debate. The methodology deployed for this study is limited in addressing this “platform bias” issue. Still, at its core the results of this study are about the effectiveness of social experiments like *Plandemic*, and future studies should develop knowledge regarding political actors behind such social experiments in a mutually beneficial development next to the “platform bias” debate.

The rapid hype cycle also adds a smaller window for when *Plandemic* related disinformation was salient in public discourse. Given that this case study exemplifies a rising and receding series of spikes in popularity around the *Plandemic* release date, and that the data was gathered a few months after June 10th, 2020, it is likely that this approach might have excluded a certain volume of relevant tweets about *Plandemic* after the period of data collection.

Similar studies have also emphasized polarization within conspiracy theory networks as important phenomena's (56, 59, 60). On the contrary, we observed few opposing voices in the dataset and the changes seen in topic categories are consistent with *Plandemic*'s general message. Given the lack of polarization in the current dataset, opposition to *Plandemic* may either be excluded from the dataset or be prevalent only after June 10th. After the release of the documentary, the current network shows only two clusters of tweets that had appeared. The two clusters were <20 nodes each and voiced clear opposition to the documentary's message. The graph layout algorithm relegated these two small clusters from the core network far outside the boundaries of the figures and it is for this reason the clusters are not visible in the figures. One the one hand, it can be reasoned that this relegation is an artifact of the echo-chamber phenomenon so prevalent in social media communities: the two clusters simply had no connections to the core network. On the other hand, if we define the *Plandemic* discourse as consisting of supporting and opposing voices, it is important to note that the sample size of this study only represents the proportions of Twitter users that voiced support for the *Plandemic*'s message and is not intended to reflect the proportion of supporters and opposers for the overall *Plandemic* discourse. One way to address this limitation is to utilize the unsupervised machine learning approaches as done so by, for instance, Haupt et al. in their study of a movement opposing public health measures during the pandemic. The current study is limited to NVIVO queries which involved hand-picking exact words and frequently occurring concepts. Unsupervised topic modeling and natural language processing may detect patterns within the content of the dataset that are possibly overlooked by human eyes, and therefore include a broader range of topics which may include other opposing voices.

## Data Availability Statement

The datasets presented in this study can be found in online repositories. The names of the repository/repositories and accession number(s) can be found below: https://tweetsets.library.gwu.edu/.

## Author Contributions

SN has queried, collected, cleaned, and analyzed all research data. All authors listed have made a substantial, direct and intellectual contribution to the work, and approved it for publication.

## Conflict of Interest

The authors declare that the research was conducted in the absence of any commercial or financial relationships that could be construed as a potential conflict of interest.

## Footnotes

^1^*Misinformation* denotes shared information that is false, regardless of any intention to mislead. Misinformation can take a range of forms within the varied and complex circumstances propagated by shifting configurations of actors, media channels, and geographies. The source of misinformation—be it a person or social media account—may be unaware that the information is false, whether due to erroneous understanding of causality, incorrect interpretation, a commonly shared but tenuous assumption, or a simple misreading of fact (3).

^2^*Disinformation* signifies a subset of misinformation. It refers to false information—such as fabricated news and manipulated content—shared by a source who holds the *intent to mislead*. For example, since the 1980's an ExxonMobil-funded network of programs and spokespeople (most notably Myron Ebell) have sought to disguise climate-science denial as a legitimate alternative to the realities of global warming. By publicizing discredited studies and presenting deliberately cherry-picked data, the campaign has successfully obfuscated public discourse with their misleading messages about global warming. The campaign's prolonged efforts and effects reverberate to this day in public discourse and attitudes towards climate change (5).
